# Supplementary material for: Radiomics and artificial intelligence in malignant uterine body cancers: Protocol for a systematic review
Source: PLoS One. 2022 Jun 8;17(6):e0267727. doi: 10.1371/journal.pone.0267727 (PMC9176798; doi:10.1371/journal.pone.0267727)
Supplement: Attachment — Submitted filename: Response to Reviewers.docx [file pone.0267727.s004.docx]

**Summary**

The paper proposes a study protocol to perform systematic reviews for radiomics and AI used for uterine body cancers (UBC) that are represented by endometrial carcinoma (EC) and uterine

sarcoma (USa).

**Publication criteria**

A completed [PRISMA-P checklist](http://prisma-statement.org/Extensions/Protocols) must be provided as a supporting information (SI) file: **Yes**

Study Protocols must also comply with general *PLOS ONE* [criteria for publication](https://journals.plos.org/plosone/s/criteria-for-publication) and in addition you should:

- include the word “Protocol” in your Title: **Yes**
- include a detailed description of the planned study in the Materials and Methods section.This should provide sufficient methodological detail for the protocol to be reproducible and replicable. Your description should cover all relevant and applicable facts and hypothesis, including:
  - the aim, design, and settling: **Yes**
  - the sample size calculation: **No**
  - how data saturation will be determined (for qualitative studies): **Yes**
  - the characteristics of participants e.g., inclusion and exclusion criteria, sample selection criteria, variables to be measured, randomization and blinding criteria (where applicable), and how informed consent will be obtained: **Yes, but doesn’t involve participants but involve inclusion and exclusion criteria for radiomics and AI articles**
  - how materials will be selected and used e.g., where and how they will be sourced, the processes, interventions, or comparisons to be used, the outcomes to be measured, and when and how they will be measured: **Yes**
  - the data management plan: **Yes**
  - safety considerations: **Not required**
  - the type of data and statistical analyses to be used: **Yes**
  - the status and timeline of the study, including whether participant recruitment or data collection has begun: **No**
  - where and when the data will be made available. See our [Data Availability policy](https://journals.https//journals.plos.org/plosone/s/data-availability) for more: **No**
- include an analysis of preliminary or pilot data, only if it is necessary to support the feasibility of the study or as a proof of principle. This is optional: **No**
- optionally add any other SI files, figures or tables that elaborate or authenticate the protocol: e.g., any reporting checklists applicable to your study type: **No**

Dear Editor, Thank you for sending me the reviewers' comments and I thank the reviewers for taking the time to make our protocol better. We will answer the questions point by point.

**Major Issues**

- Add the planned timeline for the study to meet publication criteria

We added the sentence “We expect to complete and publish the analysis by June 2022”

- Add how the data used for the proposed protocol will be made available to meet publication criteria (authors mention information will be extracted using Excel 2013 so that can be possibly be made available).
- Thanks for the comment. We included the excel sheet as supplementary table 2 (line 172)

**Minor Issues**

- Identification of studies through Scopus seems to be missing in Figure 1

Thanks for the comment, Figure 1 has been modified according to the suggestion

- It will be helpful to have a preliminary analysis as an exemplar of the use of this proposed protocol for a systematic review of radiomics and AI

We added a description of the planned analysis (line 190)

“Based on the data extracted, the overall quality of the included studies will be critically evaluated based on the “Quality Assessment of Diagnostic Accuracy Studies” tool (QUADAS-2), assessing the four standard domains (patient selection, index test, reference standard, and flow and timing). Technical aspects of the included studies will be also analysed; in this context type of techniques (MRI, CT or PET), type of segmentation adopted and how the predictive model was generated (i.e using radiomics, machine learning (ML), or deep learning (DL). We are also particularly interested in analyse the main purpose of the study (i.e for staging purpose or for lesion characterization or for survival prediction).”

- Line 42 (mentions aim of this systematic review), 102 (mentions purpose of this systematic review), 218 (mentions aim of this systematic review) but it technically should be “aim/purpose of this protocol for systematic review” since the paper has not conducted a systematic review yet and is proposing a protocol for it.

Thank you, now Lines 42, 102, 218 have been changed as requested

- Add references to briefly talk about traditional radiomics (such as Gillies et al 2016 and others) which will link to AI radiomics
- Thank you for the comment we added the follows sentences about the radiomics use in line X page Y and the subsequent references are included line 194 page 9: “The radiomics process can be divided into distinct steps such as image acquisition and reconstruction, image segmentation, features extraction and qualification, analysis, and model building [8]. Quantitative image features based on intensity, shape, size or volume, and texture offer information on tumor phenotype and microenvironment (or habitat) that is distinct from that provided by clinical reports, laboratory test results, and genomic or proteomic assays”.

**Other Comments**

- The proposed protocol can be useful and be applied to perform systematic reviews for other types of cancers.

Thanks for the comment, we definitely hope that our protocol could be useful for other specialty and be cited by other authors
